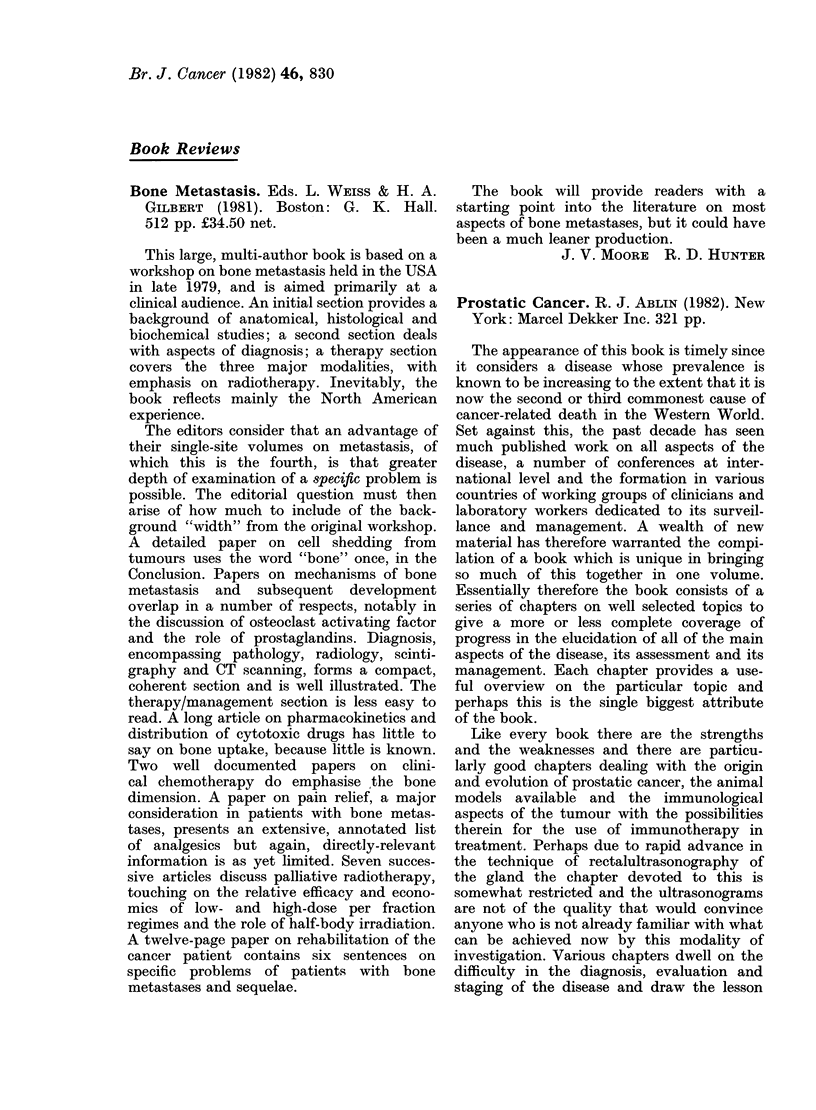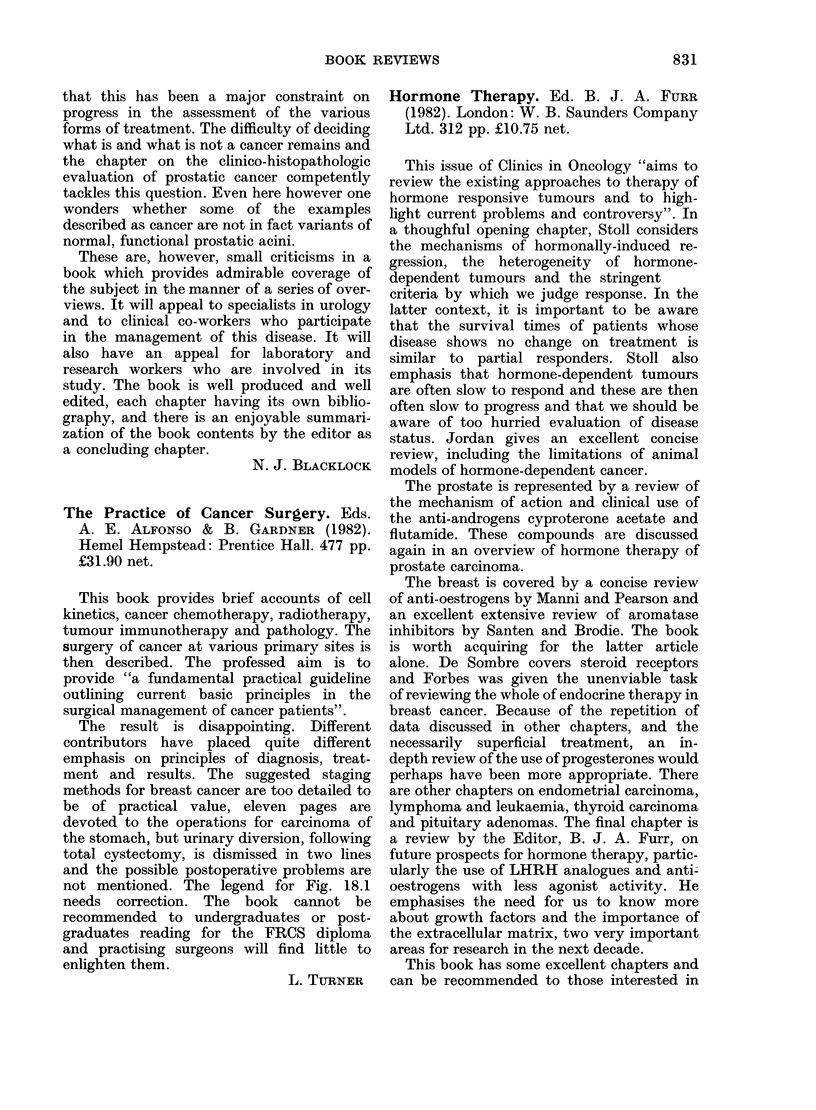# Prostatic Cancer

**Published:** 1982-11

**Authors:** N. J. Blacklock


**Prostatic Cancer.** R. J. Ablin (1982). New York: Marcel Dekker Inc. 321 pp.

The appearance of this book is timely since it considers a disease whose prevalence is known to be increasing to the extent that it is now the second or third commonest cause of cancer-related death in the Western World. Set against this, the past decade has seen much published work on all aspects of the disease, a number of conferences at international level and the formation in various countries of working groups of clinicians and laboratory workers dedicated to its surveillance and management. A wealth of new material has therefore warranted the compilation of a book which is unique in bringing so much of this together in one volume. Essentially therefore the book consists of a series of chapters on well selected topics to give a more or less complete coverage of progress in the elucidation of all of the main aspects of the disease, its assessment and its management. Each chapter provides a useful overview on the particular topic and perhaps this is the single biggest attribute of the book.

Like every book there are the strengths and the weaknesses and there are particularly good chapters dealing with the origin and evolution of prostatic cancer, the animal models available and the immunological aspects of the tumour with the possibilities therein for the use of immunotherapy in treatment. Perhaps due to rapid advance in the technique of rectalultrasonography of the gland the chapter devoted to this is somewhat restricted and the ultrasonograms are not of the quality that would convince anyone who is not already familiar with what can be achieved now by this modality of investigation. Various chapters dwell on the difficulty in the diagnosis, evaluation and staging of the disease and draw the lesson that this has been a major constraint on progress in the assessment of the various forms of treatment. The difficulty of deciding what is and what is not a cancer remains and the chapter on the clinico-histopathologic evaluation of prostatic cancer competently tackles this question. Even here however one wonders whether some of the examples described as cancer are not in fact variants of normal, functional prostatic acini.

These are, however, small criticisms in a book which provides admirable coverage of the subject in the manner of a series of overviews. It will appeal to specialists in urology and to clinical co-workers who participate in the management of this disease. It will also have an appeal for laboratory and research workers who are involved in its study. The book is well produced and well edited, each chapter having its own bibliography, and there is an enjoyable summarization of the book contents by the editor as a concluding chapter.

N. J. BLACKLOCK